# Supplementary material for: Multiparametric Classification of Non-Muscle Invasive Papillary Urothelial Neoplasms: Combining Morphological, Phenotypical, and Molecular Features for Improved Risk Stratification
Source: Int J Mol Sci. 2022 Jul 23;23(15):8133. doi: 10.3390/ijms23158133 (PMC9330009; doi:10.3390/ijms23158133)
Supplement: Supplementary file 1 [file ijms-23-08133-s001.zip › ijms-1819103-supplementary.pdf]

International Journal of *Molecular Sciences*

MDPI

Montes-Mojarro et al

Supplemental information

Supplemental Table S1. AmpliSeq Custom Panel for the analysis of non-muscular invasive UPC

Gene symbol	Transcript	Position (GRCh37/hg19)	Exon(s)	Amplicons*
ERCC2	NM_000400.4	chr19: 45,854,887 - 45,873,423	CDS	41
FGFR3	NM_000142.4	chr4: 1,795,662 - 1,808,989	CDS	38
PIK3CA	NM_006218.4	chr: 178,916,614 - 178,952,152	CDS	46
PTEN	NM_000314.8	chr10: 89,624,227 - 89,725,229	CDS	22
STAG2	NM_001042749.2	chrX: 123,156,478 - 123,234,447	CDS	66
TP53	NM_000546.5	chr17: 7,572,927 - 7,579,912	CDS	22

CDS: coding sequence. Total coverage: 99.2 %. *The amplicon lengths range between 125-175bp.

Supplemental Table S2. Baseline clinical characteristics and follow up (n = 45)

	N	PUNLMP (n = 8)	LG-UPC (n = 23)	HG-UPC (n = 14)
Baseline characteristics				
Age of first cystoscopy	45	68.5 (13.1)	73.3 (9.0)	81.4 (12.1)
		n (%)	n (%)	n (%)
Gender	45			
Male n (%)		7 (87.5%)	16 (69.6%)	10 (71.4%)
Female n 8%)		1 (12.5%)	7 (30.4%)	4 (28.6%)
Staging	45			
pTa n (%)		8 (100%)	22 (95.7%)	9 (64.3%)
pT1 n (%)		0 (0%)	1 (4.3%)	5 (35.7%)
Presence of detrusor muscle in the histological slides	44	4 (50%)	15 (65.2%)	11 (84.6%)
**Immediate therapy after diagnosis*(n=44)**				
TURB En-bloc 1/0	44	0 (0%)	2 (8.7%)	1 (7.7%)
Immediate intravesical instillation of MMC	44	4 (50%)	6 (26.1%)	2 (15.4%)
Re-TURB	44	0 (0%)	7 (30.4%)	6 (46.2%)

**Further therapy during the clinical course due to recurrence**				
Further BCG or MMC instillations	44	0 (0%)	4 (17.4%)	6 (46.2%)
Number of instillations	44			
0 n (%)		8 (100%)	19 (82.6%)	7 (53.8%)
1 n (%)		0 (0%)	3 (13%)	3 (23.1%)
2 n (%)		0 (0%)	0 (0%)	1 (7.7%)
3 n (%)		0 (0%)	0 (0%)	1 (7.7%)
6 n (%)		0 (0%)	1 (4.3%)	1 (7.7%)
Cystectomy	44	0 (0%)	1 (4.3%)	3 (23.1%)
Re-TURB	44			
0 n (%)		1 (12.5%)	7 (30.4%)	4 (30.8%)
1 n (%)		4 (50%)	7 (30.4%)	2 (15.4%)
2 n (%)		2 (25%)	6 (26.1%)	5 (38.5%)
3 n (%)		1 (12.5%)	2 (8.7%)	1 (7.7%)
4 n (%)		0 (0%)	1 (4.3%)	1 (7.7%)

Supplemental Table S3. All mutations detected

ID	Gene (NM_ID)	cDNA	Protein (aa)	VAF	Varsome	CADD PHRED (score)	Polyphen 2 (score)	SIFT (score)
**P1**	*PIK3CA* (NM_006218.4)	c.1655G>A	p.W552*	6.2%	Pathogenic	39.0	(-)	(-)
	PIK3CA (NM_006218.4)	c.2361C>G	p.D787E	11.85%	uncertain significance	23.6	possibly damaging (0.767)	Tolerated (0,60)
	PIK3CA (NM_006218.4)	c.2702G>A	p.C901Y	10.92%	Pathogenic	29.3	probably damaging (1.00)	Damaging (0.00)
	FGFR3 (NM_000142.4)	c.529C>T	p.P177S	12.65%	Uncertain significance	22.4	possibly damaging (0.910)	Damaging (0.00)
	FGFR3 (NM_000142.4)	c.1048C>T	p.H350Y	13.24%	Uncertain significance	22.4	probably damaging (0.976)	Damaging (0.00)
	FGFR3 (NM_000142.4)	c.1552G>A	p.D518N	15.81%	Uncertain significance	26.4	probably damaging (0.999)	Damaging (0.00)
	FGFR3 (NM_000142.4)	c.2315C>T	p.P772L	12.76%	Uncertain significance	25.1	probably damaging (1.000)	Damaging (0.00)
	ERCC2 (NM_000400.4)	c.1655G>A	p.W552*	10.02%	Pathogenic	51	(-)	(-)
	ERCC2 (NM_000400.4)	c.623G>A	p.S208N	10.56%	uncertain significance	22.9	benign (0.002)	Tolerated (0.25)

	ERCC2 (NM_000400.4)	c.610G>A	p.V204M	12.38%	uncertain significance	24.9	probably damaging (0.966)	Damaging (0.00)
	ERCC2 (NM_000400.4)	c.247G>A	p.V83M	14.61%	Pathogenic	29.4	probably damaging (0.994)	
	ERCC2 (NM_000400.4)	c.241G>A	p.E81K	22.22%	uncertain significance	27.1	possibly damaging (0.769)	Damaging (0.02)
**P2**	*PIK3CA* (NM_006218.4)	c.1357G>A	p.E453K	2.55%	Pathogenic	26.1	probably damaging (0.995)	Damaging (0.02)
	ERCC2 (NM_000400.4)	c.1451C>T	p.T484M	5.52%	Likely pathogenic	27.6	probably damaging (0.995)	Damaging (0.00)
**P3**	*PTEN* (NM_000314.8)	c.383A>G	p.K128R	28.96%	Pathogenic	26.6	probably damaging (0.99)	Damaging (0.00)
	FGFR3 (NM_000142.4)	c.1118A>G	p.Y373C	37.19%	Likely Pathogenic	23.0	probably damaging (0.99)	Damaging (0.01)
**P4**	*FGFR3* (NM_000142.4)	c.1118A>G	p.Y373C	46.85%	Likely Pathogenic	23.0	probably damaging (0.99)	Damaging (0.03)
**P6**	*PTEN* (NM_000314.8)	c.814C>T	p.H272Y	10.38%	Likely Pathogenic	27.1	(-)	Damaging (0.00)
		c.274G>C	p.D92H	21.96%	Pathogenic	28.5	(-)	Damaging (0.00)
		c.860C>G	p.S287*	23.06%	Pathogenic	38.0	(-)	(-)
	STAG2 (NM_001042749.2)	c.2317C>T	p.Q773*	28.7%	Pathogenic	39.0	(-)	(-)
	FGFR3 (NM_000142.4)	c.746C>G	p.S249C	16.58%	Likely Pathogenic	25.2	probably damaging (1.00)	Damaging (0.05)
**L1**	*FGFR3* (NM_000142.4)	c.746C>G	p.S249C	54.08%	Pathogenic	25.2	probably damaging (1.00)	Damaging (0.05)
	PIK3CA (NM_006218.4)	c.1633G>A	p.E545K	9.48%	Pathogenic	30.0	probably damaging (0.991)	Damaging (0.00)
	ERCC2 (NM_000400.4)	c.131C>T	p.S44L	36.34%	Likely pathogenic	32.0	probably damaging (0.992)	Damaging (0.00)
	PIK3CA (NM_006218.4)	c.3129G>A	p.M1043I	13.51%	pathogenic	23.1	benign (0.025)	Damaging (0.02)
	FGFR3 (NM_000142.4)	c.625C>T	p.Q209*	7.66%	pathogenic	49.0	(-)	(-)
**L9**	*FGFR3* (NM_000142.4)	c.746C>G	p.S249C	30.68%	Pathogenic	25.2	probably damaging (1.00)	Damaging (0.05)

	PIK3CA (NM_006218.4)	c.1357G>A	p.E453K	33.92%	Pathogenic	23.1	Possibly damaging (0.806)	Tolerated (0.19)
	PIK3CA (NM_006218.4)	c.1633G>A	p.E545K	34.45%	Pathogenic	30.0	probably damaging (0.991)	Damaging (0.00)
	ERCC2 (NM_000400.4)	c.1267G>A	p.D423N	48.33%	uncertain significance	24.1	benign (0.00)	Tolerated (0.16)
**L12**	*FGFR3* (NM_000142.4)	c.1108G>T	p.G370C	32.87%	pathogenic	13.5	Benign (0.006)	Tolerated (0.08)
	TP53 (NM_000546.5)	c.321C>A	p.Y107*	6.16%	pathogenic	34.0	(-)	(-)
**L14**	*ERCC2* (NM_000400.4)	c.256G>C	p.E86Q	12.07%	Likely Pathogenic	26.0	probably damaging (0.998)	Damaging (0.00)
**L15**	*FGFR3* (NM_000142.4)	c.746C>G	p.S249C	44.78%	Likely Pathogenic	25.2	probably damaging (1.00)	Damaging (0.05)
	STAG2 (NM_001042749.2)	c.2026-1G>A	splice site	89.58%	Pathogenic	35.0	(-)	(-)
**L17**	*FGFR3* (NM_000142.4)	c.746C>G	p.S249C	7.68%	Likely Pathogenic	25.2	probably damaging (1.00)	Damaging (0.05)
	FGFR3 (NM_000142.4)	c.742C>T	p.R248C	36.88%	Pathogenic	27.3	probably damaging (1.00)	Damaging (0.05)
	STAG2 (NM_001042749.2)	c.460G>C	p.E154Q	39.99%	Likely Pathogenic	31.0	probably damaging (0.972)	Damaging (0.01)
	STAG2 (NM_001042749.2)	c.462_462+2delGGT	splice site	32.63%				
	PIK3CA (NM_006218.4)	c.1624G>A	p.E542K	43.14%	Pathogenic	31.1	probably damaging (0.995)	Damaging (0.02)
**L18**	*FGFR3* (NM_000142.4)	c.746C>G	p.S249C	36.44%	Likely Pathogenic	25.2	probably damaging (1.00)	Damaging (0.05)
	PIK3CA (NM_006218.4)	c.1357G>A	p.E453K	40.87%	Pathogenic	26.1	probably damaging (0.995)	Damaging (0.02)
	PIK3CA (NM_006218.4)	c.1624G>A	p.E542K	43.49%	Pathogenic	31.1	probably damaging (0.995)	Damaging (0.02)
	STAG2 (NM_001042749.2)	c.3085C>T	p.Q1029*	85.13%	(-)	41.0	(-)	(-)
**L19**	STAG2 (NM_001042749.2)	c.2925-1G>C	splice site	81.52%	Pathogenic	35.0	(-)	(-)

	FGFR3 (NM_000142.4)	c.746C>G	p.S249C	78.65%	Likely Pathogenic	25.2	probably damaging (1.00)	Damaging (0.05)
**L20**	*ERCC2* (NM_000400.4)	c.256G>C	p.E86Q	53.9%	Likely Pathogenic	26.0	probably damaging (0.998)	Damaging (0.00)
	TP53 (NM_000546.5)	c.730G>T	p.G244C	76.4%	Pathogenic	29.7	probably damaging (1.00)	Damaging (0.00)
	FGFR3 (NM_000142.4)	c.746C>G	p.S249C	36.03%	Likely Pathogenic	25.2	probably damaging (1.00)	Damaging (0.05)
**L21**	*FGFR3* (NM_000142.4)	c.746C>G	p.S249C	49.93%	Likely Pathogenic	25.2	probably damaging (1.00)	Damaging (0.05)
**L23**	*FGFR3* (NM_000142.4)	c.746C>G	p.S249C	17.29%	Pathogenic	25.2	probably damaging (1.00)	Damaging (0.05)
	PIK3CA (NM_006218.4)	c.1638G>C	p.Q546H	22.11%	Pathogenic	22.2	probably damaging (0.996)	Tolerated (0.62)
**L24**	*FGFR3* (NM_000142.4)	c.746C>G	p.S249C	47.32%	Likely Pathogenic	25.2	probably damaging (1.00)	Damaging (0.05)
	ERCC2 (NM_000400.4)	c.737C>T	p.S246F	44.07%	Likely Pathogenic	28.6	benign (0.318)	Damaging (0.00)
	STAG2 (NM_001042749.2)	c.1017+3_1017+11del	splice site	54.77%	Uncertain significance	(-)	(-)	(-)
	STAG2 (NM_001042749.2)	c.1117-1G>A	splice site	5.9%	Pathogenic	33.0	(-)	(-)
	STAG2 (NM_001042749.2)	c.2635A>G	p.M879V	89.55%	Uncertain significance	22.6	(-)	Tolerated (0.08)
	FGFR3 (NM_000142.4)	c.746C>G	p.S249C	49.8%	Likely Pathogenic	25.2	probably damaging (1.00)	Damaging (0.05)
**L26**	*FGFR3* (NM_000142.4)	c.1118A>G	p.Y373C	29.88%	Likely Pathogenic	23.0	probably damaging (0.994)	Damaging (0.00)
**L27**	*FGFR3* (NM_000142.4)	c.746C>G	p.S249C	35.11%	Pathogenic	25.2	probably damaging (1.00)	Damaging (0.05)
**L28**	*FGFR3* (NM_000142.4)	c.746C>G	p.S249C	12.25%	Pathogenic	25.2	probably damaging (1.00)	Damaging (0.05)
	FGFR3 (NM_000142.4)	c.1150T>C	p.F384L	53.35%	benign	20.8	Benign (0.003)	Tolerated (0.37)
**L31**	*PIK3CA* (NM_006218.4)	c.1357G>A	p.E453K	6.2%	Pathogenic	26.1	probably damaging (0.995)	Damaging (0.02)

	FGFR3 (NM_000142.4)	c.1118A>G	p.Y373C	5.9%	Likely Pathogenic	23.0	probably damaging (0.994)	Damaging (0.00)
	ERCC2 (NM_000400.4)	c.1451C>T	p.T484M	4.72%	Likely pathogenic	27.6	probably damaging (0.995)	Damaging (0.00)
**H32**	*FGFR3* (NM_000142.4)	c.746C>G	p.S249C	14.22%	Pathogenic	25.2	probably damaging (1.00)	Damaging (0.05)
	PIK3CA (NM_006218.4)	c.1258T>C	p.C420R	38.72%	pathogenic	24.7	probably damaging (0.997)	Damaging (0.04)
**H33**	*FGFR3* (NM_000142.4)	c.746C>G	p.S249C	53.26%	Likely Pathogenic	25.2	probably damaging (1.00)	Damaging (0.05)
**H34**	*ERCC2* (NM_000400.4)	c.1816G>A	p.E606K	31.79%	Pathogenic	27.7	probably damaging (1.00)	Damaging (0.00)
	PIK3CA (NM_006218.4)	c.1252G>A	p.E418K	38.37%	Pathogenic	33.0	Possibly damaging (0.940)	Tolerated (0.19)
	TP53 (NM_000546.5)	c.736A>G	p.M246V	79.1%	Pathogenic	24.2	probably damaging (1.00)	Damaging (0.00)
**H36**	*FGFR3* (NM_000142.4)	c.742C>T	p.R248C	31.8%	Pathogenic	27.3	probably damaging (1.00)	Damaging (0.05)
**H37**	*PTEN* (NM_000314.8)	c.484G>T	p.D162Y	69.78%	Likely Pathogenic	28.6	probably damaging (0.999)	Damaging (0.00)
	TP53 (NM_000546.5)	c.742C>T	p.R248W	74.04%	Pathogenic	27.2	(-)	Damaging (0.00)
**H40**	*STAG2* (NM_001042749.2)	c.436C>T	p.R146*	13.25%	Pathogenic	35.0	(-)	(-)
**H41**	*PIK3CA* (NM_006218.4)	c.1633G>A	p.E545K	46.78%	Pathogenic	30.0	probably damaging (0.991)	Damaging (0.00)
	FGFR3 (NM_000142.4)	c.746C>G	p.S249C	88.54%	Likely Pathogenic	25.2	probably damaging (1.00)	Damaging (0.05)
	TP53 (NM_000546.5)	c.916C>T	p.R306*	92.63%	Pathogenic	37.0	(-)	(-)
**H44**	*PTEN* (NM_000314.8)	c.802G>A	p.D268N	3.04%	Likely pathogenic	32.0	benign (0.146)	Damaging (0.01)

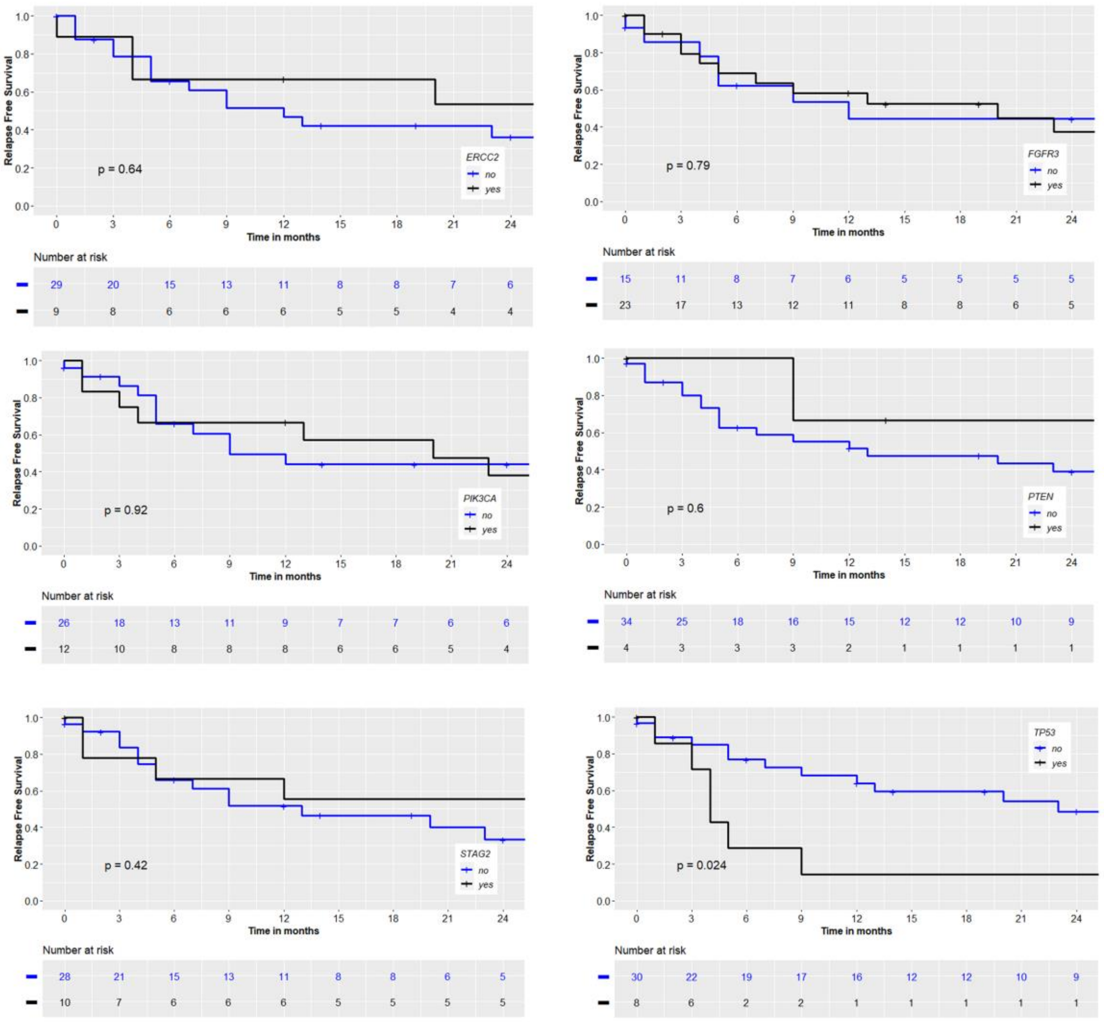

Supplemental Figure S1. Relapse-free survival using mutational status of *ERCC2, FGFR-3, PIK3CA, PTEN, STAG2, and TP53.* Kaplan-Meier analysis of cases carrying a mutation (black) compared to cases wild type (blue). The p-value is depicted in every plot.

Supplemental Table S4. Univariate analysis (proportional cox regression)

Variable	β	HR	95% CI	P value
Age first cystoscopy	-0.001	0.99	0.95 – 1.05	0.977
Female gender	0.053	1.06	0.41 – 2.69	0.911
Staging				
pTa		1		
pT1	-0.761	0.47	0.15 – 1.45	0.189
Presence of detrusor muscle in the histological slides	0.384	1.47	0.60 – 3.59	0.400
**Immediate therapy after diagnosis**				
TURB En-bloc	-0.390	0.68	0.09 – 5.07	0.704
Immediate intravesical instillation of MMC 0	-0.192	0.83	0.34 – 2.02	0.674
Re-TURB	1.086	2.96	1.21 – 7.23	0.017
**Further therapy during the clinical course due to recurrence**				
Further BCG or MMC instillations	0.193	0.82	0.79 – 1.86	0.375
**Consensus diagnosis**				
PUNLMP		1		
LG-UPC	-0.058	0.94	0.30 – 2.98	0.921
HG-UPC	0.772	2.16	0.66 – 7.08	0.202
**Cluster (two groups)**				
1		1		
2	0.905	2.47	1.07 – 5.72	0.035
**Cluster (three groups)**				
1		1		
2	0.426	1.53	0.56 – 4.16	0.403
3	0.997	2.71	0.98 – 7.53	0.056

Supplemental Table S5. Multivariable model-1

**Variable**	**β**	**HR**	**95% CI**	**P value**	**Shoenfeld residuals**
**Consensus diagnosis**					0.22
PUNLMP		1			
LG-UPC	-0.139	0.87	0.26 – 2.97	0.824	
HG-UPC	0.599	1.82	0.49 – 6.83	0.374	
Staging					
pTa		1			
pT1	-0.349	0.71	0.20 – 2.48	0.589	
Therapy	-0.156	0.86	0.33 – 2.21	0.747	

Supplemental Table S6. Multivariable model-2

Variable	β	HR	95% CI	P value	Shoenfeld residuals
**Cluster (two groups)**					0.10
**1**					
**2**	0.834	2.302	0.97 – 5.48	0.059	
**Staging**					
**pTa**					
**pT1**	-0.502	-0.606	0.19 – 1.95	0.401	
**Therapy**	-0.185	0.831	0.34 – 2.01	0.682	
